# Exploring the need for a clinical decision support system for deprescribing - A qualitative interview study

**DOI:** 10.1016/j.rcsop.2025.100574

**Published:** 2025-02-02

**Authors:** Aryoutha Asmar Talani, Tora Hammar, Ylva Böttiger

**Affiliations:** a Department of Clinical Pharmacology, County Council of Östergötland/Region Östergötland, Linköping, Sweden; b eHealth Institute, Department of Medicine and Optometry, Faculty of Health and Life Sciences, Linnaeus University, Kalmar, Sweden; c Department of Medical and Health Sciences, Faculty of Health Sciences, Linköping University, Linköping, Sweden

**Keywords:** Clinical decision support system, deprescribing, Elderly population, Polypharmacy

## Abstract

**Background:**

Deprescribing (i.e., the process of discontinuing an inappropriate medication) requires time, knowledge, and care, but there is a lack of education, support, and guidelines for this important clinical task. A clinical decision support system (CDSS) aims to influence the quality of care by combining structured medical knowledge with patient-specific information to generate recommendations.

**Objective:**

The objective was to examine the need to develop a CDSS for drug deprescribing. Furthermore, this study aimed to examine the obstacles to deprescribing and potential users' requirements for a CDSS for deprescribing.

**Methods:**

The qualitative design consisted of semistructured interviews with physicians (*n* = 10) in Sweden from different disciplines, including geriatrics, primary care and internal medicine. The interviews were conducted using a predefined guide containing multiple questions about any challenges related to deprescribing and the perceived need for a CDSS. A qualitative content analysis was performed to analyse the empirical data.

**Results:**

The interviews provided several aspects of the difficulty of deprescribing medicines. The structure and usability of the CDSS knowledge database in clinical practice needs to be ensured from the outset. Physicians needs fast, simple and up-to-date information filtered, summarized and synthesized from reliable sources. The information should preferably be integrated into pre-existing electronic health record.

**Conclusion:**

There is a need to develop a CDSS for deprescribing*.* There is little, if any, guidelines or support for deprescribing, which is regarded as a large obstacle. The current findings contribute to further knowledge regarding the perspective of physicians when deprescribing medication.

## Highlights

• Swedish physicians acknowledge several barriers to deprescribing, including the lack of readily available information on when and how to taper drugs.
• There is a positive attitude among physicians in Sweden towards using a clinical decision support system for deprescribing.
• A clinical decision support system must be user-friendly and not require more time than necessary to obtain relevant and reliable information.

## 1 Background

“Deprescribing” can be defined as a process that involves identifying and reducing the dose or discontinuing medications that have the potential to cause harm when the risk–benefit ratio for the treatment is no longer beneficial.^1^, ^2^, ^3^ Deprescribing can lead to a cost reduction for drug-related healthcare, i.e., adverse drug effects.^4^^,^^5^ Most of the time, it does not pose a problem to discontinue a drug, if the treatment is not necessary, or if the risks are considered greater than the benefits. Withdrawal symptoms may occur when drug treatment is stopped abruptly. These symptoms can vary in severity but are often harmless and transient, and can be managed by tapering the dose.^6^ However, withdrawal symptoms can also be misinterpreted as disease symptoms, leading to continued treatment.^7^

The results from previous research indicate that more knowledge, experience and courage are required to end an ongoing drug treatment than to start a new treatment.^6^^,^^8^ In addition, there is a lack of support and clinical guidelines for deprescribing.^3^ Information on how to deprescribe treatments gently and adequately is seldom available in the literature or guidelines, nor is it provided by pharmaceutical companies. A lack of time can also influence decisions regarding deprescribing.^9^^,^^10^ Good mutual trust between physicians and patients is required.^3^ From the patient's perspective, the decision to deprescribe can create anxiety, most often from the belief that they need every drug on their medication list, and that deprescribing a medicine would mean the loss of something important to them.^11^

A clinical decision support system (CDSS) combines structured medical knowledge with patient-specific information to generate recommendations, aimed at influencing the quality of care at the individual level.^12^ The CDSS consists of several parts: a clinical knowledge database, and algorithms and rules to combine patient-related information.^13^ A CDSS can be integrated either into an electronic health record or work as a stand-alone service.^14^ The information must be of high quality and presented in a predetermined manner, to ensure the accuracy and security of the system.^15^ The use of CDSS when prescribing medicines has been shown to improve health and medical care (e.g., by reducing the number of incorrect medications and increasing efficiency in patient care).^16^^,^^17^ In Sweden, electronic prescriptions are integrated with electronic health records.^18^ There is currently no CDSS for deprescribing available in Sweden.

The aim of this study was to explore the perceived need for and expectations regarding a CDSS for deprescribing by Swedish physicians. This research was conducted to answer questions such as: what difficulties do physicians experience with deprescribing, what kind of support do they use today, and what would be expected from a CDSS?

## 2 Materials and methods

### 2.1 Ethical consideration

Since the interviews covered respondents' perspectives on questions related to their work, and no sensitive or health related information was registered in the study, an approval from the Ethical Review Authority was not needed. Data were still handled, analyzed and presented in a way that no individuals could be identified, and informed consent was collected from all participants.

### 2.2 Study design

This was a qualitative study involving 30 to 60-min, semistructured, individual interviews with ten physicians from different clinical settings. The participants selected the time and location for the interviews. Due to geographical distance, some interviews were conducted online via Zoom. A semistructured interview guide (Additional file 1) was developed and used. The interview guide was developed to focus on challenges associated with deprescribing and how participants felt about using CDSS in the future. The interviews were conducted between May 2020 and July 2022. All participants were informed of the study aim before interviews were conducted, and only one researcher (AAT) attended for each interview. To obtain different perspectives from the informants, a broad representation was chosen in terms of experience, age and field of work. An inclusion criterion was that all participating physicians had at least one year of work experience in the profession. The participants consisted of three females and seven males. They had different work experiences: three residents, one intern, and six specialists. Regarding field of work, seven participants specialized in primary health care, two in geriatrics, and one in internal medicine. Inclusion continued until no new perspectives emerged from the interviews, i.e., when saturation was reached. Some of the participants in the study were selected with the help of snowball sampling, meaning that participants recommended other physicians be included. One of the authors (AAT) interviewed all ten physicians, either face-to-face or in a video meeting. The interviews were audio recorded and transcribed.

### 2.3 Analysis

Qualitative content analysis was used to categorize and describe the material from the interviews by identifying, coding, and categorizing basic patterns or themes in the empirical material.^19^ From each interview, meaning-bearing units were identified and further reduced into shorter condensed units, which were then coded by the author AAT. All coding was done by hand. Finally, the codes were grouped into categories and a total of six categories related to the study topic were formed by AAT and then agreed by all authors. Examples of qualitative content analysis showing meaningful units, their condensation and categories are given in Table 1. The categories reflected the central message of the interviews and constituted the manifest content of the texts.^19^ A few quotes (Table 2) were selected from each category to clarify what the informant mediated and for the credibility of the results.^20^ Microsoft Excel was used to organize the codes and categories.

**Table 1:** Example of qualitative content analysis showing meaningful units, their condensation, and categories.

Meningful units	Condensed meaningful units	Code	Categories
When it comes to lipid-lowering treatment, I have an idea of how to deprescribe, but deprescribing medications you do not work with on a daily basis can be difficult. This applies also for increasing or decreasing medication.	You know how to deprescribe medications that you are used to. Medicines you don't have experience with on a daily basis are more difficult to deprescribe, reduce or increase.	Medicines that you do not work with on a daily basis can be difficult to deprescribe.	Knowledge of deprescribing

**Table 2:** Selected quotes from physicians for each of the seven categories.

**Challenges of deprescribing** *“If you give the right information, it often goes well. It does happen that some patients are hospitalized due to drug- related problems. In that case, it is easier to make a decision and persuade the patient to stop treatment, but some people say, do not touch my meds.” (Physician 1)* *“After all, it is more difficult to persuade a patient who has had sleeping pills for 20 years, it rarely works and usually you have to swallow your pride and hand out a new prescription.” (Physician 2)* *“I had a patient who had been on epilepsy medication for several years but had been seizure free for a long time. The drug concentration was measured and was far too low to have an effect. Most likely, the medicine had no effect, and we could end the treatment. Moreover, the patient was informed and felt calm with the decision.” (Physician 5)* **Time-consuming process** *“Sometimes it is easiest to renew the prescription, especially if you are short on time, even though you do not know the patient, or fully understand the indication. Unfortunately, you do not have time to prioritize such matters.” (Physician 4)* *“We do not want to step on anyone's toes. We physicians have a responsibility, and if we see an inappropriate prescription, it must be deprescribed. It is always more difficult to go against the decision of a colleague who is responsible for the patient and has prescribed a medicine that you yourself think is inappropriate. You will not deprescribe it unless it is obviously wrong”. (Physician 2)* **Knowledge of deprescribing** *“The strategy that I use is that you follow up, do it slowly, and hope that you have a common understanding with the patient. Furthermore, it is good to have some knowledge of how medicines work and their elimination half-lives. I have no other strategy than that”. (Physician 7)* **Physicians need for support.** *“It would have been good to have greater access to pharmacists or FASS with information about deprescribing. Otherwise, I generally google if there is an unknown substance. However, I do not read scientific articles that are time-consuming, and I do not have that time”. (Physician 2)* *“I would say that it is as much the responsibility of other physicians to look at the patients' whole medication list as it is for general practitioners who works at health center”. (Physician 3)* **CCDSs design** *“Make it easy and accessible; if it is in XX [name of EHR], I will use it. I prefer not to* log *in and out from many places, you do not have time.” (Physician 6)* *“Yes, interactions, pregnancy alerts and breastfeeding alerts are useful. The good thing is that there is not only a recommendation but also a description”. (Physician 1)* *“I think this is a system that most people would accept very easily. The right information at the right time, no one would find it difficult. FASUT, is not the easiest book in the world to read, but otherwise good information.” (Physician 2)* *“In primary care, we do not get extra time for extra tasks, and everything must be accommodated within the half hour that the patient is with us. A system with many bugs that consumes much time becomes something negative quite quickly.” (Physician 3)* **National intervention** *“I feel that national campaigns tend to get stuck. Although time passes and interest has grown among staff, the region chooses to wait until the next update. If a national intervention is to be made, it should be when it is actually launched.” (Physician 7)* *“Just as [I think] the medication list and the electronic health record should be national, I also think that it should apply to all medication management throughout Sweden, whether it concerns deprescribing or prescribing.” (Physician 3)* **The patients access to CDSS** *“A patient may get in touch and wonder why it is a warning flag for this prescription,* etc. *Then, you will have to be on the phone a lot and explain, and it can get messy.” (Physician 1)*

## 3 Results

Content analysis of the interviews (n = 10) resulted six main categories. Below, each of the main categories is described. Table 2 shows relevant quotes from the physicians.

### 3.1 Challenges of deprescribing

The physicians agreed that they found deprescribing more difficult than prescribing. Psychotropic drugs, antidepressants, analgesics and sleeping pills were perceived to be more difficult to deprescribe than, for example, drugs for hyperlipidemia. The willingness of patients to stop treatment is important, and closely related to physician–patient relationships and the time needed for dialogue. There were also considerations as to whether the decision to deprescribe was correct from an ethical perspective. The evaluation of drug efficacy can be particularly difficult in cases of long*-*standing treatments with unclear indications. There were mixed opinions on whether it was more difficult to stop drugs prescribed by other physicians, especially from another speciality, and when the drug was recently prescribed. This also applied to colleagues with whom they worked with on a daily basis, when they felt less inclined to end the prescription.

### 3.2 Time-consuming process

Almost all the physicians expressed that deprescribing was not prioritized during medical appointments, especially under time pressure. A recurring comment was that deprescribing was time-consuming, requiring planning for both dose tapering and follow-up visits. In primary health care, a digital service for prescription renewal may be available, making it more difficult to have a patient dialogue regarding deprescribing. One reason for not ending another physician's prescription was a lack of knowledge about the drug, in combination with a lack of time for reading on the subject.

### 3.3 Knowledge of deprescribing

All interviewees stated that there is no lack of information about medicines but that it would be desirable to have the information collected in one place. Information from web pages, guidelines, books, and contact with a colleague or a clinical pharmacist was widely used. Google was also used as a source of information, even though the physicians were aware of the risks of not receiving reliable information. All the physicians agreed that there was little mention of deprescribing during medical training, except for a general recommendation to stop long-standing medications. Although there was much discussion about polypharmacy during medical training, the physicians felt that there was a lack of specific teaching about deprescribing. This knowledge was acquired during clinical practice. The physicians had access to a book, “FASUT”, at their workplace, but not all of them used it. Some physicians did not have time to read the book or could not find a copy.

### 3.4 Physicians' need for support

Physicians expressed the importance of having readily accessible and reliable support for deprescribing, especially during medication reconciliations. The lack of information on deprescribing in the Swedish medicines information portal was mentioned by several physicians. Almost all the physicians preferred digital services over books. Some of the physicians mentioned that they have access to clinical pharmacists for consultations regarding deprescribing concerns.

The majority of physicians interviewed agreed that it was easier to make changes to the treatment when the medication list was complete and up to date. The physicians also agreed on the importance of communication between different health care providers around the patient, concerning both prescribing and deprescribing.

### 3.5 CDSS design

The physicians were generally positive about using a CDSS for deprescribing. The geriatricians especially pointed out the need for support, and expected that a CDSS would save considerable time. Ideally, the CDSS should be integrated into the existing EHR for easy access. Today, physicians often work with several systems in parallel, and find it difficult to log on to, and keep track of all systems.

The physicians were careful to point out that usability is crucial, with a minimum of unnecessary warnings and pop-ups to disturb the workflow. Additionally, the system will not be used if it has many bugs or is too complicated.

In addition to ready-made deprescribing schedules for each substance, there was also a desire for information on how to switch medications. Physicians also wanted general information on the pharmacokinetic properties of medicines. All agreed that they preferred precise suggestions rather than general reflections on deprescribing. Physicians mentioned the importance of continuous updates of the CDSS. Almost everyone agreed that a national system would be an advantage.

### 3.6 Patient access to the CDSS

There were mixed opinions regarding patient access to the CDSS. The physicians thought it was good to let the patient take part in their care plan and to have access to information contained in the CDSS. However, they pointed out that it was important that the patient was aware that the suggestions by their physicians may differ from those of the CDSS. Another thing mentioned was that it may confuse the patient if they have access to a CDSS but lack medical competence.

## 4 Discussion

In line with previous studies, this investigation showed that deprescribing is perceived as more difficult than prescribing.^9^ The participants expressed lack of time, knowledge and guidelines as obstacles to deprescribing. Patients taking multiple medicines often have multiple physicians, which further adds to the difficulties.^21^ A lack of continuity in the patient–physician relationship was also mentioned as a factor that may lead to unreflective renewal of prescriptions rather than re-evaluation and possible deprescribing of treatments. The physicians in this study were aware of the need to reduce the dose gradually to avoid withdrawal symptoms when stopping certain medicines but pointed out that it is time-consuming to write out and follow up on tapering plans. They also pointed out that there is a hesitancy to deprescribe medication that has been prescribed by a specialist at the hospital. This is similar to the findings of Alrawiai (2023).^3^

Patient-related obstacles to deprescribing included perceived or actual substance dependence, as well as anxiety related to stopping long-standing treatments, even though the indication or effect may be unclear. Previous research has concluded that trust and communication are important tools for deprescribing, and that it is important that patients have confidence in their physician.^4^^,^^11^ This was also evident from the physician's views in the present study.

In general, physicians expressed a positive attitude towards the use of a clinical decision support system for deprescribing, especially in view of the lack of available information on how to stop drugs elsewhere. Although physicians have access to books, they prefer digital tools for easy access to information.

The main expectations regarding a deprescribing CDSS expressed by the interviewees were that it must be user friendly, time saving and included into the workflow, preferably as an integrated feature of the electronic health record. These results are consistent with previous research showing that a CDSS should be considered user friendly.^10^^,^^17^ Although a tool can be perceived as having many advantages, time-consuming tools are less likely to be used, which Rieckert et al. (2019)^22^ also described. During the interviews, a noted concern was that the CDSS would provide disturbing pop-ups for less important information, which can lead to users clicking away all warnings immediately without reading them. This is congruent with findings in previous studies that CDSSs provide too many warnings and can result in alert fatigue, meaning that physicians may miss or neglect relevant warnings that need to be addressed.^23^

Despite recognizing the need for patient involvement in treatment decision making, the physicians interviewed in this study suggested that a deprescribing CDSS should primarily be a tool and work instrument for the prescriber, and opinions regarding the patient's access to the CDSS varied in the group. However, other studies support the involvement of patients, also in the access and use of a CDSS (there is evidence that patient involvement facilitates deprescribing).^24^^,^^25^ A review of the literature showed that many patients would like to be able to check their medications themselves^26^ and be more involved in the withdrawal process.^10^ This was also shown by Alrawiai (2023), whose findings suggested that building a trusting relationship with the patient reduced the main obstacles for deprescription.^3^

The focus of this study was on prescription drugs for patients of all ages. Therefore, it is valuable to investigate, prior to the introduction of a new CDSS, multiple areas where deprescription may become relevant. For example, older patients with multiple chronic conditions. Thus, the involvement of physicians who treat elderly patients in the development of the CDSS at an early stage will likely contribute to reducing the barrier to deprescribing.^17^ General practitioners (GPs) can also be seen as the ideal health care professionals to participate in the development process, as they know the patients' medical histories and what medications they are currently taking, and have previously used.^3^

Another aspect that may be valuable to study is the physician's concerns with deprescribing drugs, which was not mentioned in the results section but should be highlighted. One must consider the psychological factors that might affect physicians to deprescribe, as there is a fear that the patient will die or be harmed in some way, which Alrawiai (2023)^3^ also described.

A strength of this study lies in the selection of participants (different specialists from both hospitals and health centres), which can contribute to providing different perspectives for deprescribing. However, in a smaller sample, several perspectives can be missed, which exhibits as a limitation of this study. To have greater credibility and wider application, a larger sample of health care professionals is needed for interviews. Another limitation of this study is its focus on the Swedish healthcare system, which restricts the generalizability of the findings to other systems. It is important that fundamental work should be done before the implementation of such a CDSS. Future CDSSs should also be evaluated after implementation to receive suggestions for improvement.

## 5 Conclusion

Swedish physicians acknowledge several barriers to deprescribing, including the lack of readily available information on when and how to taper medicines, and are generally positive towards using a computerized decision support system for this task, as long as it is user friendly and time saving. This research has demonstrated the value of a CDSS from the physician's perspective and can further contribute to the development of a CDSS that is both highly relevant and provides reliable information. To conclude, as in any other clinical situation, physicians need fast, simple, and up-to-date information on deprescribing, which is filtered, summarized, and synthesized from reliable sources by clinical and preclinical experts, to be integrated into electronic health records.

## Funding

Not applicable.

## Ethics approval and consent to participate

Participants provided informed consent before participating in the interview study.

## Consent for publication

Consent for publication was obtained from the study participants.

## CRediT authorship contribution statement

**Aryoutha Asmar Talani:** Writing – review & editing, Writing – original draft, Visualization, Validation, Software, Methodology, Investigation, Formal analysis, Data curation. **Tora Hammar:** Writing – review & editing, Writing – original draft, Validation, Supervision, Resources, Project administration, Methodology, Investigation, Formal analysis, Data curation, Conceptualization. **Ylva Böttiger:** Writing – review & editing, Writing – original draft, Visualization, Validation, Supervision, Software, Resources, Project administration, Methodology, Investigation, Funding acquisition, Formal analysis, Conceptualization.

## Declaration of competing Interest

The authors declare that they have no known competing financial interests or personal relationships that could have appeared to influence the work reported in this paper.

## Data Availability

The datasets (audio files and written transcripts) generated and/or analyzed during the current study are not publicly available due to privacy considerations but available from the corresponding author on reasonable request.

## Appendix A Interview guide

Introductory questions

- Age?

- Professional title?

- Work experience?

- How long have you worked at your current workplace?

1. Do you think it is difficult to deprescribe?

- If yes/no, why? Give examples?

- Difficult to deprescribe someone else's prescription? Feel free to develop!

- What are the most difficult things related to deprescribing? Why?

- Can you tell us what kind of access you have/had to information regarding deprescribing?

- Where do you obtain your knowledge from?

- Do you know the book, FASUT? Do you use the book?

- Do you feel that you lack information about how to deprescribe?

2. Have you previously participated in training courses/seminars regarding deprescribing?

- During your schooling or ongoing work?

3. How do you stay up to date on new information regarding, e.g., new drugs and guidelines?
4. What are your thoughts on a new CDSS specifically for deprecribing?

- Do you see any benefit?

- Would you use it?

- Requests for functionality?

6 National intervention in the CDSS. What do you think?
7 Should patients have access to the CDSS, what do you think?
8 Is there anything you would like to add?
